# Life cycle assessment of nutrient recovery strategies from domestic wastewaters to quantify environmental performance and identification of trade-offs

**DOI:** 10.1038/s41598-024-54060-6

**Published:** 2024-02-14

**Authors:** Carla Mae Pausta, Pradip Kalbar, Devendra Saroj

**Affiliations:** 1 Centre for Environmental Health and Engineering (CEHE), School of Sustainability, Civil and Environmental Engineering, University of Surrey, Guildford, GU2 7XH UK; 2 Centre for Urban Science and Engineering (CUSE), Indian Institute of Technology Bombay, Powai, Mumbai, 400076 India

**Keywords:** Life cycle assessment, Nutrient recovery, Wastewater, Septage, Water quality, Environmental impact, Environmental monitoring, Pollution remediation, Sustainability

## Abstract

Increase in anthropogenic activities proliferated the consumption of resources such as phosphorus; and increase the adverse environmental impacts especially eutrophication on water resources such as lakes. Nutrient recovery from domestic wastewaters to produce a fertiliser has been explored to address these challenges in the context of a sustainable circular nutrient economy. Life cycle assessment (LCA) was performed to holistically assess the impacts of integrating a nutrient recovery system on wastewater and water resource management using Laguna de Bay, Philippines as the geographical boundary. The inventory was developed based on the results of the emerging nutrient recovery reactor operations and the application of the recovered fertiliser on the agricultural crops. The LCA results for the proposed scenario showed environmental benefits of about 83.6% freshwater eutrophication, 102.5% terrestrial ecotoxicity, 26.9% water consumption, 100.7% mineral resource scarcity, while the global warming potential is 95.4% higher than the baseline scenario. Results imply policy review for septage management, system optimisation, and evaluation of alternative methods of wastewater management, in terms of life cycle thinking and sustainability across the globe.

## Introduction

The traditional linear supply chains brought about by the industrial revolution and economic development have ensued massive extraction of resources, and significant increase in waste generation resulting in the decline of planetary health^1^. This affects transgressions in the global food security and increase in adverse environmental impacts that are interconnected with water-nutrient management and agricultural systems in hotspot regions, mainly in Asia^2^. However, the improvement of the quality of water resources and nutrient pathways would require complex problem-solving and collective efforts from scientists, policy makers, and the rest of the society^3^. Hence, a paradigm shift from the linear flow model to integrated industrial ecosystem is required, that adapts the circular economy approach and life cycle thinking^4^. Nutrient recovery and recycling from waste streams, particularly wastewater, has been considered an efficient driver in closing the loop towards sustainable development and resilience in water-nutrient management^5^. This would also particularly address the urgent challenges in phosphorus resource depletion and eutrophication in the context of agri-food systems, clean water, and sustainable sanitation^6^.

Eutrophication is one of the leading environmental issues, that is as complicated to control and manage as climate change^7,8^. Developing countries in Asia, such as Philippines, are greatly affected with eutrophication due to high population density and lack of appropriate sewage and septage treatment systems^9^. In fact, the biggest inland water body in the Philippines, Laguna de Bay (Laguna Lake), is already experiencing the effects of eutrophication with regular occurrences of mass fish mortalities^10^. This affects 21.4 million people who rely on the lake as their major source of food, water, and livelihood. This prompted the Philippine government to amend its existing policies to update the wastewater effluent quality standards as most of the wastewater treatment plants do not involve nutrient removal yet^11,12^. Consequently, about 84% of the households in the Philippines use septic tanks that do not conform to standards often resulting to overflow of raw septage, further contributing to environmental pollution^13^. Hence, appropriate treatment of septage must also be prioritised while exploring the opportunity to utilise resource-oriented technologies for onsite sanitation systems to recover valuable nutrients.

Nutrient removal is essentially a part of wastewater treatment systems, but nutrient recovery from wastewater has also been explored to promote circular economy in the context of phosphorus (P). Recovery of P from waste streams could substitute 17–31% of the phosphate-rock based P fertilisers by 2030^14^. In Europe, there have been changes in environmental regulations that require the recovery of P from wastewater for agricultural use^15^. For this purpose, struvite recovery systems are being preferred and widely studied because of the process efficiency and production of a high value fertiliser, thus also promoting economic sustainability^16^. Struvite (NH_4_MgPO_4_·6H_2_O) is a slow-release fertiliser that can be recovered from wastewater through the addition of magnesium salts at alkaline condition^17^. Although some countries are already integrating nutrient recovery^18,19^, the Philippines has just recently started monitoring nutrient pollution of water resources from point sources, and nutrient recovery is yet to be explored.

Nutrient recovery from wastewater could provide new perspectives on lake water management that could stop nutrient pollution at household-level and potentially shift global problem-solving from end-of-pipe to process-integration solutions^20^. Recently, a pilot-scale nutrient recovery reactor has been installed at a local farm to provide a proof-of-concept on the potential of septage for resource recovery in the context of circular economy^21^. Although the technology is established, the application of this system would require further sustainability evaluation, as stakeholders would necessitate systematic and quantitative assessment of the risks and impacts^22^. Other countries especially in Europe have explored the economic and environmental benefits of nutrient recovery but this is highly contextualised based on the local setting^23^. Moreover, the integration would incur additional use of energy and chemicals, and produce emissions to the environment^24,25^. Therefore, there is a need to objectively evaluate the potential of a nutrient recovery system in the Philippines.

Life Cycle Assessment (LCA) covers cradle-to-grave approach to quantify the environmental performance of a certain product or system throughout its life cycle, from raw material acquisition, production, utilization, end-of-life, waste treatment, recycling and disposal^26^. LCA has been used as a holistic approach to evaluate the environmental impacts of several wastewater sludge management systems in a localised setting^27,28^. LCA of wastewater treatment systems should be conducted on a site-specific basis for improved assessment on impacts specifically on eutrophication and toxicity-related impact categories due to the spatial effects and characteristics of the emissions involved^29,30^. Various LCA studies on nutrient recovery from wastewater showed more environmental benefits for eutrophication and resource extraction^19,24,31–33^. However, without having an integrated energy recovery, the global warming potential will be higher due to the life cycle emissions of added chemicals and energy^19,24,34^. Consequently, LCA studies are typically built with massive data from primary, and secondary sources, and some arbitrary assumptions. Uncertainty analysis could be performed to provide robustness in the inventory and LCA results. Most LCA studies do not incorporate uncertainty analysis, hence, further investigation may be needed to validate confidence in the results^35^.

In this study, LCA was utilised to systematically evaluate the impacts of adapting a nutrient recovery system for sustainable management of water-nutrient resources and wastewater. Nutrient recovery has never been implemented as a full-scale system for septage (i.e. human waste) management at decentralised scale. A pilot-scale nutrient recovery reactor study carried out in the Philippines has produced significant results to provide technical evaluation of the potential of sewage and septage as a nutrient resource^21^. Moreover, utilising septage as resource for nutrient recovery is an opportunity, that is barely explored, to treat the major domestic wastewater discharge while providing an alternative fertiliser that could incur savings to local farmers and avoid further agricultural water run-offs. Previous LCA studies focus on various wastewater feedstock such as sewage and source-separation facilities for urine and faeces^36^, but LCA has never been performed for nutrient recovery from decentralised systems such as septic tanks. The novel aspect of this research is the LCA of nutrient recovery from septage considering a specific geographical boundary to evaluate the realistic environmental conditions of the possible scenarios.

In this paper, three scenarios are considered encompassing the geographical boundary surrounding the Laguna Lake. Scenario 1 covers the current situation with respect to domestic wastewater treatment for both sewage and septage. Scenario 2 is triggered from the changes in government policies for effluent quality, thus involves integration of nutrient removal technology in sewage treatment plants (STP) for compliance, while septage is remained unmonitored. Scenario 3 includes the proposed integration of nutrient recovery system to both STPs and septic tanks. The system boundary is extended to the application of the recovered fertiliser to agriculture and treated wastewater discharge to nearby water resources including Laguna Lake. The life cycle impact assessment was carried out using ReCiPe method. Uncertainty analysis was performed in order to provide higher confidence in the LCA results. Sensitivity analysis was used to evaluate the effects of varying the relevant parameters on the impact results. This LCA study provides holistic quantification of the environmental benefits and burdens brought about by the integration of proposed nutrient recovery system. The LCA results provide insights for policy development and baseline justification for nutrient recovery process integration in domestic wastewater treatment towards sustainability and circular economy.

## Methods

### Goal and scope definition

The life cycle assessment (LCA) was carried out using the ISO 14,040:2006 standard procedure comprising four stages: goal and scope, life cycle inventory, life cycle impact assessment, and interpretation^26^. The goal of this study is to evaluate the environmental impacts of integrating a nutrient recovery system to conventional sewage treatment plants (STP), and to onsite sanitation systems (i.e. septic tanks) for the improvement of water quality in lakes. The functional unit is 1 m^3^ of domestic wastewaters produced by the population within the geographical boundary. The geographical boundary includes the cities and provinces surrounding Laguna Lake, serving 21.4 million population and 5.3 million households^37^. The inventories cover the operational phase for all scenario. The construction and end-of-life phase are not included. The domestic wastewaters in this study cumulatively comprises 23% sewage from 1.2 million households connected to STPs, and 77% of septage from 4.1 million households that use septic tank. The statistics on population, and households were taken from the Philippine Statistics Authority (PSA). The data on water consumption, social class, and septage produced were taken from the Philippine Institute for Development Studies^37,38^. All statistics and derived data are presented in the Table S1 of the supplementary information.

### Scenarios

**Figure 1:**
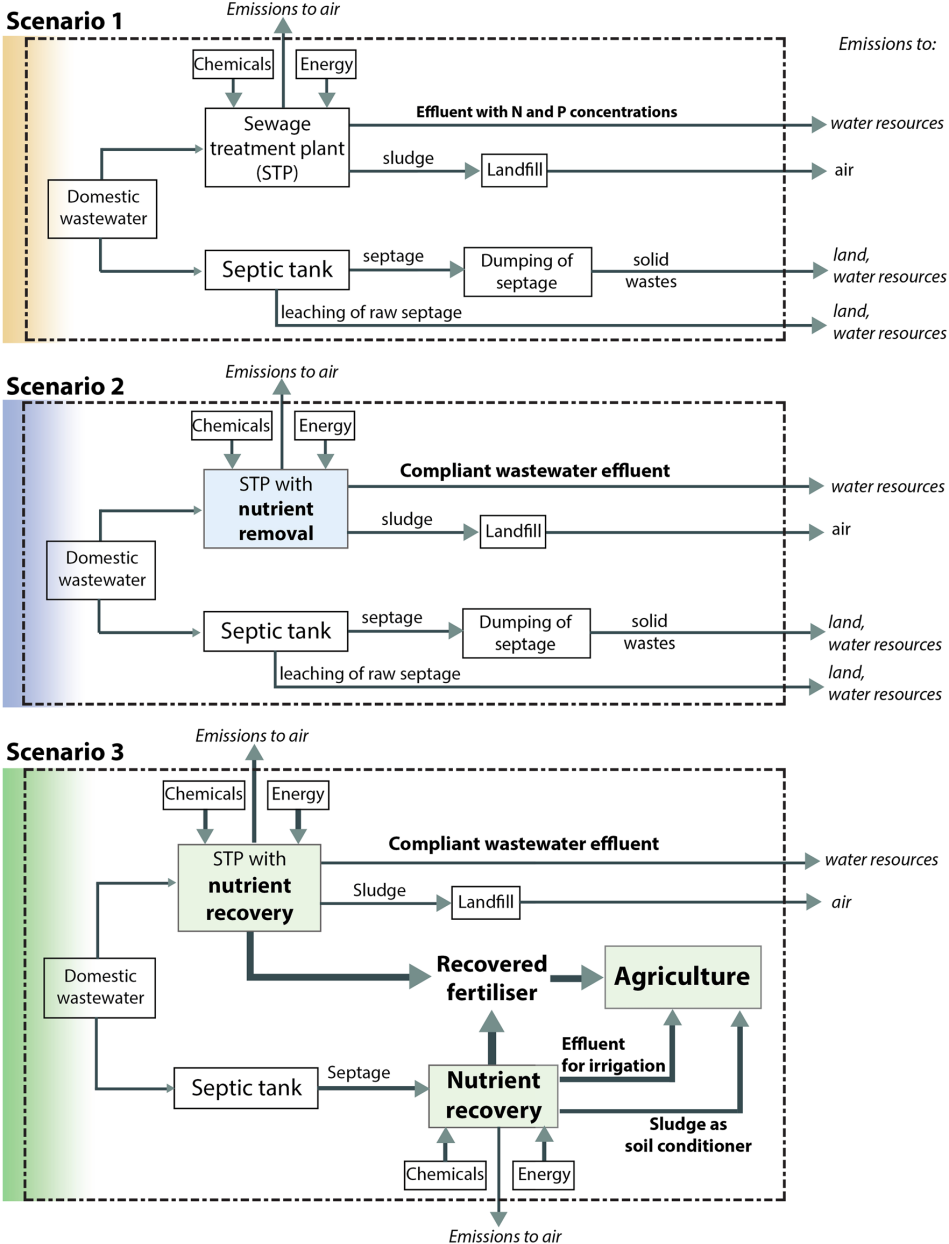
System boundary for the three scenarios.

The system boundary starts with the domestic wastewaters being discharged as sewage and as septage, shown in Fig. 1. The septage is transported to different disposal and treatment methods for each of the scenarios, while the sewage is directly transported through piping systems to centralised STPs. Scenario 1 is considered as the baseline or current scenario wherein conventional activated sludge (CAS) is being utilised as the wastewater treatment system. Metro Manila has centralised STPs wherein the wastewater undergoes CAS treatment or utilises sequencing batch reactors, but the effluent discharged to water bodies is not yet compliant with the government regulations, Department of Environment and Natural Resources Administrative Order (DAO) 2016–08 and DAO 2020–19, in terms of ammonia, nitrate and phosphate concentrations^11,12^. The provinces Laguna, Cavite, Rizal, and Batangas mostly use septic tanks wherein only 5% of households transport their septage to STPs, while 95% of the household population de-sludge their septage then dump in unauthorised dumpsites, thus both sludge and effluent are leaching through soils, groundwater, and water bodies without proper handling and treatment^13^. Scenario 2 is considered as the required and compliant scenario that covers full integration of nutrient removal technologies for STPs, as non-compliance to DAO 2016–08 and DAO 2020–19 would entail enormous penalty costs and termination of operations. For provinces, the current scenario for septic tanks, where septage are discharged to unauthorised dumpsites, is utilised as there is no current systematic monitoring yet for individual households and communities^13^. Scenario 3 covers the integration of STPs, and septic tanks with nutrient recovery system as proposed in our previous research study^21^. A pilot-scale nutrient recovery reactor was installed in a local university farm being resided by students and faculty, wherein septage was treated through hydrolysis and nitrogen and phosphorus fertiliser was recovered as struvite through chemical precipitation. Acid hydrolysis, in particular, was utilised as a pre-treatment method prior to the chemical precipitation to release the phosphorus and nitrogen into soluble forms and to kill the pathogens^21^. The recovered fertiliser was applied to crops as an alternative to commercial fertiliser. Moreover, the waste sludge produced was analysed to be suitable to use as supplemental fertiliser and the effluent was reused as irrigation water for crops. The inventory inputs are summarised in the process flow diagram and material balance shown in the supplementary information (Fig. S1).

### Life cycle inventory

The life cycle inventory (LCI) of the common and background economic and environmental flows are based on EcoInvent 3.0^39^ and Agrifootprint^40^ databases. The summarised LCI and the calculated avoided products for every scenario are shown in Table 1. The localised flows such as demographics, geographical boundaries, electricity mix, and transportation, were based from the official reports of PSA, LLDA, and Department of Energy (DOE). The detailed inventories are presented in the Supplementary Information Tables S1, S2, S3, and S4. The flows and emissions for Scenario 3 were taken from the results of our previous research study on the pilot-scale nutrient recovery reactor that processes both sewage from a centralised wastewater treatment plant and septage from septic tank^21^. The other data for the conventional wastewater and septage treatment were taken from other literatures as cited. The Philippine electricity mix contains 43.1% coal, 14.5% oil-based, 12.8% natural gas, and 29.5% renewable energy, of which comprises 5.4% solar, 13.6% hydroelectric, 1.6% wind, 7% geothermal, and 1.8% biomass^41^. Power requirements for the scenarios are: (1) 0.52 kWh/kg COD removed^42^, (2) 1.20 kWh/kg COD removed^42^, (3) addition of 16.72 kWh/kg recovered fertiliser produced^21^. For the chemical data, 7 kg of polymers are used per ton of dry sludge^43^. The distance of chemical suppliers to the STPs is assumed to be 60 km. For the direct emissions to air due to wastewater treatment operations, around 1.375 kg CO_2_/kg BOD removed from organic matter oxidation, 0.035 kg N_2_O–N/kg N denitrified, and 0.0125 kg CH_4_/kg COD removed^43,44^. The estimated waste sludge production in an STP is 0.22 kg/m^3^ for Scenarios 1 and 3, and 0.26 kg/m^3^ for Scenario 2^45^. The waste sludge from the septic tank for scenarios 1 and 2 with about 54.41 kg/m^3^ for each scenario is being disposed in unauthorised dumpsites by third-party haulers within the range of 60 km^13^. The sludge for Scenario 3 was utilised as a supplemental fertiliser based on its characterisation^21^. The waste sludge from STPs will be transported to landfills located within the distance of 60 km, wherein 13.4 g of methane and 35.12 g of carbon dioxide for every kg of sludge, are emitted to air^45^.

**Table 1:** Summary of the life cycle inventory for the three scenarios.

		Scenario 1 (1 m^3^)		Scenario 2 (1 m^3^)		Scenario 3 (1 m^3^)		Recovered struvite—scenario 3 input
		STP	Septic tank	STP	Septic tank	STP	Septic tank	
Functional unit		1 m^3^	1 m^3^	1 m^3^	1 m^3^	1 m^3^	1 m^3^	290 g
**Materials/fuels**								
Recovered struvite	kg	–	–	–	–	2.90		–
Polyacrylamide	kg	0.0015	–	0.0208	–	0.0015	–	–
Hydrochloric acid (30%_w_)	g	–		–		–		852
Ammonium chloride	g	–		–		–		9.32
Magnesium chloride hexahydrate	g	–		–		–		18.33
Sodium hydroxide (50%_w_)	g	–		–		–		61.957
Transportation, EURO 4	kgkm	0.0924	–	1.248	–	0.0924	–	60.24
Tap water	kg	1093.9		1093.9		1093.9		–
**Avoided products**								
Phosphate (P_2_O_5_) fertiliser	g	–		–		3540.80		58.71
Nitrogen (N) fertiliser	g	–		–		765		8.265
Potassium chloride (NPK 0-0-60)	g	–		–		518.03		–
Water	kg	–		–		383.70		–
**Electricity**								
Electricity mix, PH	kWh	9.49	–	19.98	–	19.98	–	4.85
**Emissions to air**								
Carbon dioxide	g	1880	–	1880	–	1880	–	–
Methane	g	0.21	–	0.21	–	0.21	–	–
Dinitrogen monoxide	g	–	–	17.93	–	17.93	–	–
**Emissions to water**								
BOD, Biological Oxygen Demand	g	25	1392	25	1392	25	12	-
COD, chemical oxygen demand	g	50	16,700	50	16,700	50	71	–
Suspended solids	g	50	–	50	–	50	50	–
Ammonia, as N	g	4	103.00	2	103.00	2	0.50	–
Nitrate	g	53	14.30	7	14.30	7	11.16	–
Phosphate-P	g	4	7.80	2	7.80	2	1	–
Arsenic (As)	g	–	0.07	–	0.07	–	0.01	–
Calcium (Ca)	g	–	544.25	–	544.25	–	1570	–
Cadmium (Cd)	g	–	0.07	–	0.07	–	0.001	–
Chromium (Cr)	g	–	0.35	–	0.35	–	–	–
Copper (Cu)	g	–	0.02	–	0.02	–	–	–
Iron (Fe)	g	–	207.00	–	207.00	–	0.10	–
Magnesium (Mg)	g	–	57.75	–	57.75	–	77.00	–
Manganese (Mn)	g	–	7.95	–	7.95	–	–	–
Nickel (Ni)	g	–	0.43	–	0.43	–	–	–
Lead (Pb)	g	–	1.10	–	1.10	–	0.01	–
Zinc (Zn)	g	–	43.75	–	43.75	–	0.03	–
**Emissions to soil**								
Arsenic (As)	g	–	2.25	–	2.25	–	3.50	–
Calcium (Ca)	g	–	16,300	–	16,300	–	–	–
Cadmium (Cd)	g	–	4.35	–	4.35	–	3.10	–
Iron (Fe)	g	–	20,500	–	20,500	–	6300	–
Magnesium (Mg)	g	–	2270	–	2270	–	780	–
Lead (Pb)	g	–	56	–	56	–	104	–
Zinc (Zn)	g	–	3570	–	3570	–	2310	–
Total phosphorus	g	–	8765	–	8765	–	1545	–
Total nitrogen	g	–	13,250	–	13,250	–	7650	–
Potassium	g	–	895	–	895	–	271	–
**Waste**								
Waste sludge	kg	0.22	54.41	0.26	54.41	*Avoided*		–
Transportation, EURO 4	kgkm	12.90	3265	15.60	3265			–

The inventory for scenario 3 includes avoided materials and emissions due to the recovery of fertiliser, utilisation of the by-product sludge as supplemental fertiliser or soil conditioner, and reuse of effluent as irrigation water in crops. Fertilisers recovered from wastewater has plant availability of 50% nitrogen and 70% phosphorus, as the recovered fertiliser may contain other precipitates and the chemical structures could affect the plant uptake^46^. Thus, approximately 2.85% of the recovered fertiliser in struvite form, is available for nitrogen plant uptake while 8.83% for phosphorus uptake. For every nitrogen and phosphorus recovered as fertiliser, the same amount of nitrogen and phosphorus in commercial fertilizers are avoided, hence the calculated avoided products are 202 g P_2_O_5_ /kg recovered fertiliser and 28.5 g N fertiliser/kg recovered fertiliser. Consequently, 3,540 g P_2_O_5_/m^3^ of wastewater, 765 g N fertiliser/m^3^ of wastewater, and 518 g KCl fertiliser/m^3^ of wastewater is avoided with respect to sludge utilisation in farms; and 0.384 m^3^ water/m^3^ of wastewater is avoided with respect to water reuse. The burdens brought about by the production of the avoided products are then subtracted from the impacts of producing the recovered fertiliser^47^.

### Life cycle impact assessment

The life cycle impact assessment (LCIA) was evaluated using the ReCiPe method for midpoint level of impact indicator characterisation^48^, and was processed through SimaPro v9. The midpoint damages are highlighted in this study to efficiently analyse and characterise the direct impacts of the environmental flows included in the system boundary^49^. In this way, the required improvements and optimisation requirements of the scenarios can be specifically identified. The environmental impact indicators on midpoint level are global warming potential (GWP), stratospheric ozone depletion, ionizing radiation, ozone formation-human health, fine particulate matter formation, ozone formation-terrestrial ecosystems, terrestrial acidification, freshwater eutrophication, marine eutrophication, terrestrial ecotoxicity, freshwater ecotoxicity, marine ecotoxicity, human carcinogenic toxicity, human non-carcinogenic toxicity, land use, mineral resource scarcity, fossil resource scarcity, and water consumption.

### Uncertainty analysis

The uncertainty for each scenario was evaluated using Monte Carlo to test the robustness of the LCIA results, due to the potential variability of the inputs in the inventory^50^. The simulations were performed at 95% confidence level, with 1000 iterations. Though precision increases with the number of iterations, accuracies tend to decrease, so 1,000 iterations are enough for this purpose^35,50^. The parameters that can vary based on future decisions and consequences brought about by the change in government policies and process development are struvite yield, septage characteristics, and sludge yield. It is assumed that variability of values would be about 10%, at uniform distribution, of the average values used in the inventory.

### Sensitivity analysis

Sensitivity analysis was performed to evaluate the effects of the input parameters in the LCIA results^51^. In this study, the concept of Design of Experiments (DoE) was utilised to generate regression equations that could represent and explain the effects of factors to the response^52,53^. The factors considered are the amount of recovered fertiliser and the renewable energy percentage while the response considered is the global warming potential (GWP). The nutrient recovery system can still be optimized with respect to the hydrolysis efficiency and changes in parameters. Hence, the amount of recovered fertiliser produced was treated with up to + 50% variation, that is 4.35 g recovered fertiliser for every L of septage as the high value, from the low value of 2.90 g/L that was used in the inventory. The renewable energy percentage share in the Philippine electricity grid is expected to increase from 29.5 to 50% by 2040^54^, thus the low value used was 29.5% from the inventory, while the high value is 50%. The Space Filling Latin Hypercube design was utilized as the sampling method to allow more sampling coverage with minimal runs. The DoE was performed with 20 runs and the regression equations were generated using the Design-Expert software by Stat-Ease.

## Results

### Life cycle impact assessment

The life cycle impact assessment (LCIA) results based on midpoint damage indicators are shown in Table 2, wherein scenario 3 demonstrated the highest environmental benefits in terms of water consumption, fossil resource scarcity, mineral resource scarcity, land use, human non-carcinogenic toxicity, marine ecotoxicity, freshwater ecotoxicity, terrestrial ecotoxicity, marine eutrophication, freshwater eutrophication, and stratospheric ozone depletion. Consequently, scenario 3 has the highest environmental burdens in terms of global warming potential (GWP), ionising radiation, ozone formation human health, fine particulate matter formation, ozone formation terrestrial ecosystems, terrestrial acidification, and human carcinogenic toxicity. The environmental burdens can be attributed to the energy-intensive processes and chemical addition to recover the nutrients. However, there is an urgent necessity to improve the water quality and avoid eutrophication through the reduction of nutrient discharges to the water resources.

**Table 2:** Environmental impact assessment characterisation: endpoint and midpoint level indicators.

Midpoint level impact indicators	Unit	Scenario 1	Scenario 2	Scenario 3
Global warming potential	kg CO_2_ eq	2.18	5.42	47.93
Stratospheric ozone depletion	kg CFC11 eq	2.42 × 10^–07^	4.57 × 10^–05^	-2.22 × 10^–04^
Ionizing radiation	kBq Co-60 eq	6.65 × 10^–02^	6.83 × 10^–02^	2.53 × 10^–01^
Ozone formation, human health	kg NOx eq	4.97 × 10^–03^	9.75 × 10^–03^	8.50 × 10^–02^
Fine particulate matter formation	kg PM_2.5_ eq	8.06 × 10^–03^	1.60 × 10^–02^	1.20 × 10^–01^
Ozone formation, terrestrial ecosystems	kg NO_x_ eq	5.24 × 10^–03^	1.03 × 10^–02^	8.97 × 10^–02^
Terrestrial acidification	kg SO_2_ eq	2.58 × 10^–02^	5.30 × 10^–02^	3.85 × 10^–01^
Freshwater eutrophication	kg P eq	0.73	0.73	0.12
Marine eutrophication	kg N eq	1.36	1.36	0.74
Terrestrial ecotoxicity	kg 1,4-DCB	2.33	3.60	− 93.18
Freshwater ecotoxicity	kg 1,4-DCB	8.94	8.95	4.30
Marine ecotoxicity	kg 1,4-DCB	7.85	7.86	3.33
Human carcinogenic toxicity	kg 1,4-DCB	0.46	0.46	0.64
Human non-carcinogenic toxicity	kg 1,4-DCB	104,060.95	104,061.23	67,366.26
Land use	m^2^a crop eq	9.32 × 10^–03^	1.03 × 10^–02^	− 4.20 × 10^–01^
Mineral resource scarcity	kg Cu eq	2.34 × 10^–03^	2.68 × 10^–03^	− 3.53 × 10^–01^
Fossil resource scarcity	kg oil eq	0.69	1.35	− 5.08
Water consumption	m^3^	1.10	1.10	0.80

**Figure 2:**
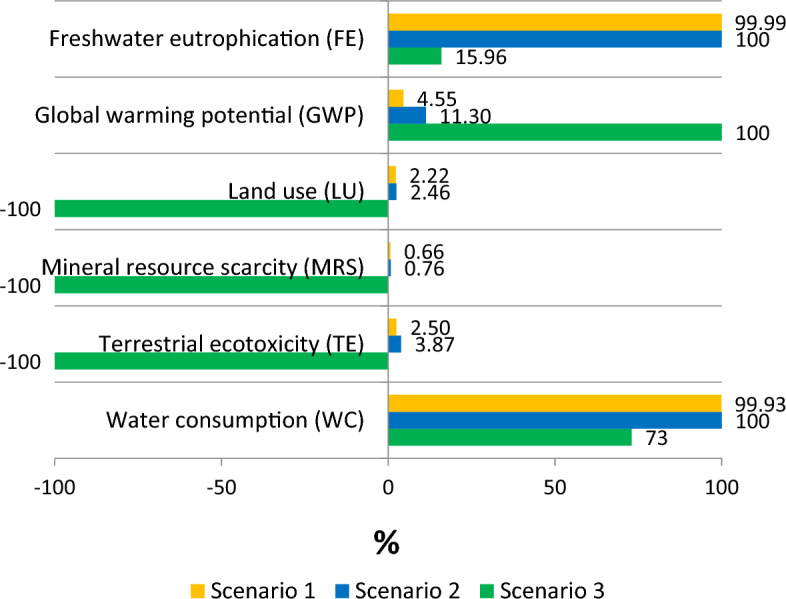
Significant life cycle midpoint level normalised results for Scenario1, Scenario 2, and Scenario 3.

The normalisation of the midpoint impacts is shown in Fig. 2, to demonstrate the relative impacts for the three scenarios, such that 100% is the highest positive value corresponding to environmental burdens, while -100% is the lowest negative value corresponding to environmental benefits. For every 1m^3^ of domestic wastewater discharge as the functional unit, Scenario 3 has about 84% less of P-eq discharge for freshwater eutrophication compared to Scenarios 1 and 2. This is highly attributed to the improvement of nutrient removal from effluent discharges and efficiency of fertiliser uptake of plants^32^. There is about 102.5% less of 1,4-DCB emissions for terrestrial ecotoxicity, due to avoided use of commercial fertilisers and avoided pesticide emissions to agricultural lands. The land use was found to be beneficial with Scenario 3, that could be mainly attributed to the avoided landfilling and unauthorised dumping of septage and waste sludge. Moreover, as there is a pressing urgency in the lack of water supply within the Laguna Lake region, Scenario 3 can provide about 26.9% of water consumption savings. The mineral resource scarcity for Scenario 3 showed 100.7% of Cu-eq savings due to the avoided use of fertiliser, hence avoiding further extraction of phosphate rock minerals. However, a vital trade-off that was hypothesised would be the GWP. In comparison to Scenario 1, Scenario 3 has produced an addition of 45.75 kg CO_2_-eq, while scenario 2 has produced about 3.24 kg CO_2_-eq more. A critical discourse should be made as GWP is an important issue that contributes to the degradation of planetary health^2^. The LCIA midpoint level results suggest that though Scenario 3 has most of the environmental advantages, there are direct emissions that needs to be addressed. This could be through optimisation of the proposed nutrient recovery system to minimise chemical and energy usage, while maximising nutrient recovery and recycling of by-products.

### Uncertainty analysis

**Figure 3:**
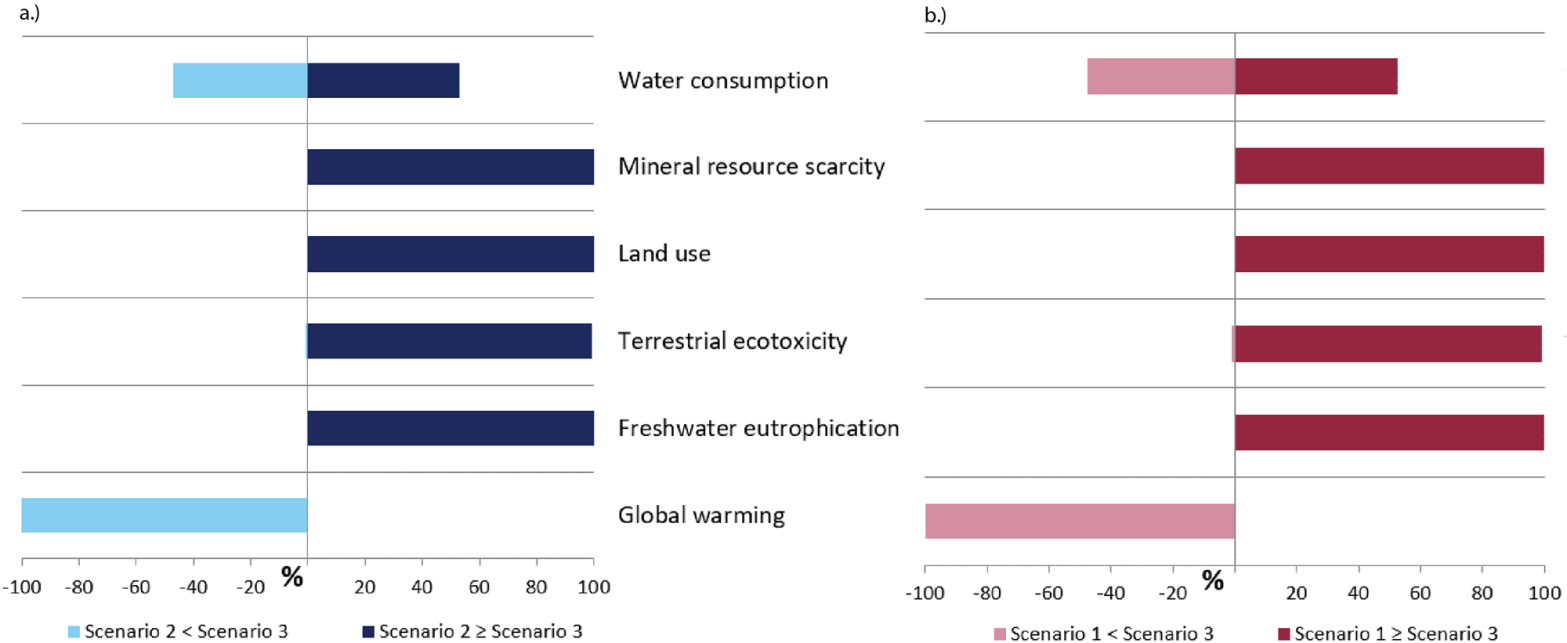
Uncertainty analysis for (**a**) Scenario 3 and Scenario 2, (**b**) Scenario 3 and Scenario 1.

The relative comparison of midpoint level indicators of Scenario 3 with Scenario 1 and Scenario 2 with respect to uncertainties at confidence level of 95% are shown in Fig. 3. In general, the relative uncertainties of Scenario 3 with Scenario 1 and Scenario 2 are similar since the LCIA results for Scenario 1 and Scenario 2 are almost the same in magnitude. Uncertainty results show that regardless of the degree of variabilities of the input parameters in the inventories, Scenario 3 will always have higher environmental benefits for mineral resource scarcity, land use, terrestrial ecotoxicity, and freshwater eutrophication, while having more environmental burdens for GWP. This means higher confidence in the LCIA results for the aforementioned indicators. For water consumption, the uncertainty of parameters showed that there is a probability of 50% deviation to the LCIA results since about 50% chance that Scenario 3 will have higher environmental burdens compared to Scenarios 1 and 2. This could be attributed to the avoided water consumption due to the reuse of wastewater effluent. The deviation in LCIA results for human carcinogenic toxicity, and ionising radiation can be attributed to the waste sludge produced for Scenarios 1 and 2, and the avoided supplemental fertiliser due to the alternative agriculture application of the by-product sludge for Scenario 3.

The inventories are based from different data sources. Conducting the uncertainty analysis provided higher confidence with the LCA results given that some inventory parameters have a certain degree of uncertainty because of internal model changes and possible data variability^50^. Additionally, the results showed insights on the robustness of the LCA model, results and data quality while considering the specific goal and scope of this study, without neglecting future development on government policies, and further optimisation of the required nutrient removal technologies and the proposed integration of nutrient recovery processes for water and wastewater management.

### Sensitivity analysis

The sensitivity analysis was performed to evaluate the effects of two factors, amount of recovered fertiliser and increase of renewable energy utilisation, on the global warming potential (GWP) impact assessment results. The summary of the runs and the results of the responses are summarised in Table 3, while the detailed calculations and inputs are presented in the Supplementary Information Table S5.

**Table 3:** Sensitivity analysis design and results.

Run	Factors				Reponses		
	Recovered fertiliser, g/L (A)		Renewable energy, % (B)		GWP_S1_, kg CO_2_-eq	GWP_S2_, kg CO_2_-eq	GWP_S3_, kg CO_2_-eq
	low	high	low	high			
	2.90	4.35	29.50	50.00			
1	3.587		33.82		2.081	5.203	60.585
2	3.129		42.45		1.878	4.775	45.194
3	4.045		30.58		2.157	5.363	73.198
4	4.274		35.97		2.030	5.096	73.947
5	3.358		39.21		1.954	4.935	52.004
6	3.968		43.53		1.852	4.721	61.553
7	4.121		47.84		1.751	4.507	61.227
8	3.663		41.37		1.901	4.823	56.824
9	3.282		50.00		1.700	4.400	43.470
10	3.816		37.05		2.005	5.042	63.240
11	3.053		38.13		1.979	4.989	46.222
12	3.511		45.68		1.802	4.614	50.795
13	3.892		31.66		2.132	5.310	68.934
14	4.350		44.61		1.827	4.668	68.391
15	3.205		34.89		2.056	5.149	51.502
16	2.976		46.76		1.776	4.561	39.535
17	2.900		32.74		2.107	5.256	46.071
18	3.434		29.50		2.183	5.417	60.113
19	4.197		40.29		1.929	4.882	68.811
20	3.739		48.92		1.725	4.454	53.003

The runs were simulated for ANOVA and the resulting regression equation for every scenario for GWP are as follows:

1 $${GWP}_{S1}=2.87-3.2\times {10}^{-5}A-0.0236B$$

2 $${GWP}_{S2}=6.88-6.7\times {10}^{-5}A-0.0496B$$

3 $${GWP}_{S3}=-16.75+28.13A-0.0499B-0.1805AB$$

where GWP_S1_, GWP_S2_, and GWP_S3_ are the GWP responses for Scenarios 1, 2, and 3, respectively; A refers to the amount of recovered fertiliser produced, B refers to the percent of renewable energy sources in the Philippine electricity mix. Based on the generated equations, Eqs. (1) and (2), Scenario 1 and Scenario 2 follows a linear model where A is a very low value since there is no recovery of fertiliser involved while B has a negative coefficient implying that the higher the renewable energy percent, the lesser is the GWP impact. The results for Scenario 3 follows a two-factor interaction ANOVA denoted by the AB term in Eq. (3). The negative coefficient shows that the higher the recovery rate and the higher the percentage share of renewable energy sources, the lesser the adverse impact with respect to GWP. This suggest the importance of nutrient recovery system optimisation and process-integration for potential energy recovery to promote the water-energy-nutrient nexus.

### Interpretation

Through this research, the environmental impacts of the current, required, and proposed scenarios in the context of the geographical boundary, Laguna Lake, requiring urgent solutions to wastewater and water-nutrient management were quantified. Retrofitting all onsite sanitation systems is not possible in the next few years since these involve massive infrastructure changes especially in places having dense population, space limitations, and lack of economic capacity. A proactive approach that would require process integration, policy improvement and execution could provide a critical and urgent solution to the challenges affecting human health, ecosystem, and resources. Moreover, the LCA tool provided an insight on the potential of the proposed nutrient recovery in terms of circular economy, as all economic flows are utilised within the system boundary^55^.

The integration of localised nutrient recovery system in communities or individual household could provide life cycle environmental benefits. Since the LCA was performed considering the geographical boundary of Laguna Lake, the long-term benefits include the improvement of the lake water quality, hence improves food and water security, among others. Moreover, the proposed scenario will increase stability in the context of agri-food systems due to the improved water and nutrient recycling within the system boundary. To further improve and develop a more sustainable nutrient recovery process in the life cycle context, the LCIA midpoint levels should be analysed as well^49^.

The decrease in freshwater and marine eutrophication, for Scenario 3 is highly attributed to the nutrient removal and recovery from all wastewater types (i.e. sewage and septage). Aside from nutrient recovery, its application to agriculture also contributed to the avoided emissions, since the recovered fertiliser releases P and N slower than the commercial and conventional fertilisers. Thus the efficiency of nutrient uptake in plants is increased, minimising further nutrient-rich agriculture run-offs, as run-offs contribute to eutrophication at the lake^8^. Additionally, the by-product sludge can also be applied in agricultural crops, contributing to avoided landfilling and indirect nutrient emissions^56^. The proposed technology has also produced an effluent that can be recycled as irrigation water for agriculture, thus incurring water consumption savings and life cycle advantages for water extraction from the lake.

The increase in GWP for the integration of nutrient recovery processes has been observed in many LCA studies^19,24,34^, due to the added chemical and energy usage^57^. Though there are avoided commercial fertilisers, it’s not enough to compensate with life cycle CO_2_ equivalent emissions within the geographical boundary, since the nutrient recovery reactor utilised in the scenario is community-based. Commercial fertilisers are processed in industrial scale, producing higher fertiliser yields with optimised energy efficiency. Moreover, the Philippine electricity grid currently is highly dependent in non-renewables sources. The government had already set a target to have at least 50% of renewable energy in the country’s power generation mix by year 2040^41^. This could make the process integration be more favourable for the decrease in GWP^58^. As this technology is the first application in this geographical context, the GWP results and sensitivity analysis in this LCA study triggers further research, optimisation and scaling up.

The current scenario (Scenario 1) and the required scenario (Scenario 2) have comparable LCIA results for most impact indicators, shown in Fig. 2. This indicates that the improvement of STPs with nutrient removal processes only provides benefits at source and surface level. Environmental damages in terms of life cycle are still present in another form (i.e. midpoint level indicators) such as increase in GWP and break-even impact results. Since the STPs are located in compact cities, integrating developments provided more environmental burdens than benefits due to added chemicals and use of energy, among others. This means that the imposed changes in the wastewater effluent quality regulations (i.e. DAO 2019) do not necessarily produce a significant improvement in the environmental life cycle. Moreover, optimisation and selection of the best nutrient removal technology should be identified to prioritise and promote an efficient nutrient recovery^59^. Even if all the STPs follow the current regulations and integrate nutrient removal, there will be no significant improvement especially for eutrophication. This could be attributed to the large percentage of septage, at around 77% of domestic wastewater, being discharged to Laguna Lake and other nearby water bodies without proper treatment, system design, and monitoring. The LCA results provides an important insight that targeting STPs is not enough as most of the untreated discharges come from septage.

Scenario 3 proposed pursuing both sewage and septage for nutrient removal and nutrient recovery, thus the life cycle environmental impacts significantly decreased in magnitude. The past years, the priority of the government has always been with sewage and industrial wastewater. Lesser to no importance at all was given to the monitoring of septage management especially for decentralised and onsite sanitation systems, for cities and communities despite having existing regulations^13^. The results of this study could change the perspective, targets, and goals of the government and other stakeholders with regards to water resource and wastewater management. The proposed scenario provides an opportunity to improve the lake water quality, decrease eutrophication, minimise fertiliser importation, and possibly boost the local economy.

### Way forward

This research clearly indicated the necessity to review the wastewater management policies, optimise the nutrient recovery from domestic wastewaters, and explore alternative solutions. Application of life cycle perspective revealed that there are trade-offs between the different environmental impacts when chemical-based resource recovery is adopted. Hence, nature-based solutions have recently emerged as an alternative to tackle sustainability and resilience issues in infrastructure^60^. Especially such solutions are more relevant for water sector. Hence, the way forward in this research is to explore nature-based and hybrid treatment systems wastewater treatment systems^61^. This includes integration of energy recovery through biological pathways, and utilisation of green chemicals that could improve yields and decrease energy consumption^62,63^. Furthermore, nature-based solutions combined with scaled decentralization may be better suited for developing countries such as Philippines than chemical-based resource recovery^64^. Considering various aspects such as higher GWP potential for chemical-based resource recovery, operational difficulty, and economic feasibility, there is need to explore other alternatives for sustainable wastewater treatment in such settings. Thus, there is a need to identify alternatives or optimise the existing systems.

Future LCA research will be performed to evaluate the potential of these alternative systems while considering the sustainability factors within the geographical boundary. Life cycle sustainability assessment will also be conducted to evaluate the socio-economic aspects. Moreover, integration of other tools such as multi-criteria decision analysis could provide a holistic understanding of the inherent decision-making trade-offs in proposing alternative systems^55,65^. Consequently, the current policies and its implementation should be reviewed with the relevant government agencies and stakeholders in order to propose necessary improvements on decentralised wastewater and septage management.

## Conclusion

Life cycle assessment (LCA) was performed to quantify the environmental impacts of the proposed integration of nutrient recovery for domestic wastewater treatment and improvement of lake water quality at Laguna de Bay, Philippines. The proposed scenario, Scenario 3, was compared with two other wastewater treatment scenarios, the current scenario (Scenario 1), the transition and required scenario (Scenario 2). Based on the life cycle impact assessment results, Scenario 3 has the least adverse environmental impacts on water consumption, fossil resource scarcity, mineral resource scarcity, land use, human non-carcinogenic toxicity, marine ecotoxicity, freshwater ecotoxicity, terrestrial ecotoxicity, marine eutrophication, freshwater eutrophication, and stratospheric ozone depletion, while having the most environmental impacts on global warming potential, ionising radiation, ozone formation human health, fine particulate matter formation, ozone formation terrestrial ecosystems, terrestrial acidification, and human carcinogenic toxicity, compared to the other two scenarios. Particularly, about 83.6% freshwater eutrophication, 102.5% terrestrial ecotoxicity, 26.9% water consumption, 100.7% mineral resource scarcity were reduced providing more environmental benefits due to nutrient removal and recovery, avoided use of commercial fertilisers, and water consumption savings. However, due to the added utilisation of chemicals and energy, the global warming potential for Scenario 3 is 95.4% higher than Scenario 1. Uncertainty analysis results show more confidence in the inventory and the impact assessment results, providing robustness of the LCA model. In general, this study provided quantified results and revealed the trade-offs between different impact categories that emerge while applying resource recovery technologies. These insights could be interpreted to aid decision-making challenges of stakeholders on the integration of nutrient recovery system on wastewater treatment facilities especially on decentralised systems, for the restoration and improvement of planetary health, and for the planet’s sustainability and resilience. Future research will focus more on sustainability assessment and identification of alternatives for resource recovery from wastewater in the context of circular nutrient economy and water-energy-nutrient nexus.

### Supplementary Information

Supplementary Information.

## Data Availability

All data generated or analysed during this study are included in this published article and its supplementary information file.
