# Supplementary material for: Life cycle assessment of nutrient recovery strategies from domestic wastewaters to quantify environmental performance and identification of trade-offs
Source: Sci Rep. 2024 Feb 14;14:3678. doi: 10.1038/s41598-024-54060-6 (PMC10867002; doi:10.1038/s41598-024-54060-6)
Supplement: Supplementary file 1 — Supplementary Information. [file 41598_2024_54060_MOESM1_ESM.pdf]

Life cycle assessment of nutrient recovery strategies from domestic wastewaters to quantify environmental performance and identification of trade-offs

Authors: Carla Mae Pausta[1], Pradip Kalbar[2], and Devendra Saroj[1]*
[1]Centre for Environmental Health and Engineering (CEHE), School of Sustainability, Civil and Environmental Engineering, University of Surrey, Guildford GU2 7XH United Kingdom
[2]Centre for Urban Science and Engineering (CUSE), Indian Institute of Technology Bombay, Powai, Mumbai, 400076, India
*Corresponding author: d.saroj@surrey.ac.uk

Supplementary Information

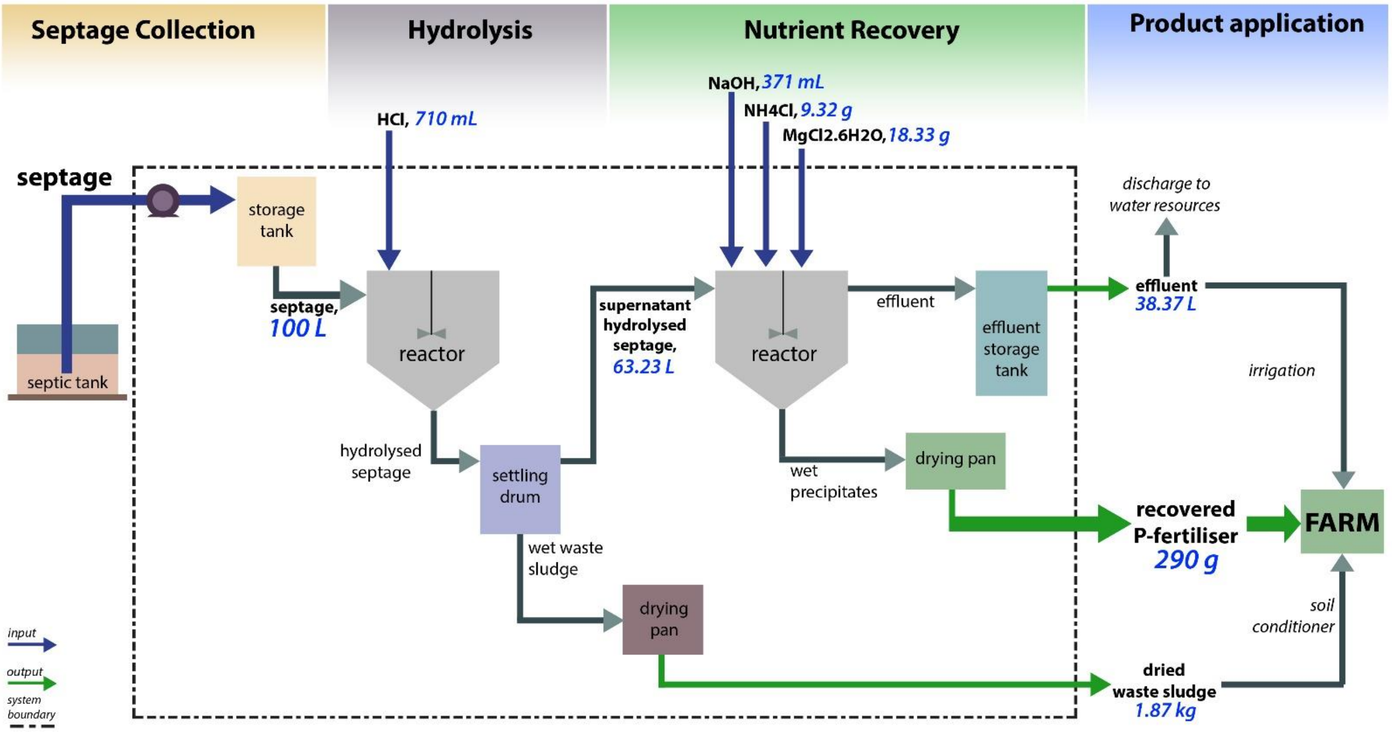

Figure S1. Process flow diagram with material balance for Scenario 3 (integration of nutrient recovery system) inventory (Pausta et al., 2023)

Table S1. Statistics on population and households, and calculated data domestic wastewater treatment routes within the geographical boundary (ie. Sewage Treatment Plants, STP; and septic tanks) (Philippines Statistics Authority, 2021)

Geographical Boundary	Population	Households	*Households connected to:*	
			Septic Tanks	STP
Metro Manila	13,484,462	3,288,893	2,302,225	986,668
Laguna	3,382,193	915,398	915,398	204,316
Rizal	3,330,143	784,402	784,402	
Cavite				
Silang	295,644	71,463	71,463	
Carmona	106,256	28,154	28,154	
General Mariano Alvarez	172,433	41,233	41,233	
Tagaytay City	85,330	22,399	22,399	
Batangas				
Tanauan City	193,936	46,680	46,680	
Malvar	64,379	17,064	17,064	
Sto. Tomas	218,500	59,686	59,686	
Quezon				
Lucban	53,091	12,692	12,692	
TOTAL	21,386,367	5,288,064	4,086,326	1,190,984
Percentage contribution of STP and septic tank				
STP, %	23			
Septic Tank, %	77			

Table S2. Water Consumption and calculated septage distribution for every social class members within the geographical boundary (Albert et al., 2018; Philippines Statistics Office, 2021)

Class	Population	Water consumption L/c/day	Water consumption L/day	Dry g feces/c/day	Dry g feces/day
Poor	12,489,638	48	599,502,639.74	38	474,606,256
Middle	8,597,320	58	498,644,532.97	38	326,698,142
High	299,409	108	32,336,186.90	28	8,383,456
Total water consumption, L	**1,130,483,359.62**				
Drinking water consumption, L/day	106,931,835				
Septic tank input					
Total water going to septic tank, L	1,023,551,525				
Feces, kg	809,688				
Total septage, kg	**1,024,361,212**				
Septage/water consumption, %	**90.61**				

Table S3. Detailed life cycle inventory for all the scenarios

		Scenario 1 (1 m^3)		Scenario 2 (1 m^3)		Scenario 3 (1 m^3)		Recovered struvite – Scenario 3 input
		STP	Septic tank	STP	Septic tank	STP	Septic tank	
**Functional Unit**		1 m^3	1 m^3	1 m^3	1 m^3	1 m^3	1 m^3	290 g
Materials/fuels								
Recovered struvite	kg	-	-	-	-	2.90		-
Polyacrylamide	kg	0.0015	-	0.0208	-	0.0015	-	-
Hydrochloric acid (30%$_w$)	g	-		-		-		852
Ammonium chloride	g	-		-		-		9.32
Magnesium chloride hexahydrate	g	-		-		-		18.33
Sodium hydroxide (50%$_w$)	g	-		-		-		61.957
Distance to chemical suppliers	km	60	-	-	-	60	-	60
Transportation, EURO 4	kgkm	0.0924	-	1.248	-	0.0924	-	60.24
Tap Water	kg	1,093.9		1,093.9		1,093.9		
Avoided Products								
Phosphate (P_2O_5) fertiliser	g	-		-		3,540.80		58.71
Nitrogen (N) fertiliser	g	-		-		765		8.265
Potassium chloride (NPK 0-0-60)	g	-		-		518.03		-
Water	kg	-		-		383.70		-
Electricity								
Electricity mix, PH	kWh	9.49	-	19.98	-	19.98	-	4.85
Emissions to air								
Carbon dioxide	g	1,880	-	1,880	-	1,880	-	-
Methane	g	0.21	-	0.21	-	0.21	-	-
Dinitrogen monoxide	g	-	-	17.93	-	17.93	-	-
Emissions to water								
BOD, Biological Oxygen Demand	g	25	1,392	25	1,392	25	12	-
COD, Chemical Oxygen Demand	g	50	16,700	50	16,700	50	71	-

Suspended solids	g	50	-	50	-	50	50	-
Ammonia, as N	g	4	103.00	2	103.00	2	0.50	-
Nitrate	g	53	14.30	7	14.30	7	11.16	-
Phosphate-P	g	4	7.80	2	7.80	2	1	-
Arsenic (As)	g	-	0.07	-	0.07	-	0.01	-
Calcium (Ca)	g	-	544.25	-	544.25	-	1,570	-
Cadmium (Cd)	g	-	0.07	-	0.07	-	0.001	-
Chromium (Cr)	g	-	0.35	-	0.35	-	-	-
Copper (Cu)	g	-	0.02	-	0.02	-	-	-
Iron (Fe)	g	-	207.00	-	207.00	-	0.10	-
Magnesium (Mg)	g	-	57.75	-	57.75	-	77.00	-
Manganese (Mn)	g	-	7.95	-	7.95	-	-	-
Nickel (Ni)	g	-	0.43	-	0.43	-	-	-
Lead (Pb)	g	-	1.10	-	1.10	-	0.01	-
Zinc (Zn)	g	-	43.75	-	43.75	-	0.03	-
Emissions to soil								
Arsenic (As)	g	-	2.25	-	2.25	-	3.50	-
Calcium (Ca)	g	-	16,300	-	16,300	-	-	-
Cadmium (Cd)	g	-	4.35	-	4.35	-	3.10	-
Iron (Fe)	g	-	20,500	-	20,500	-	6,300	-
Magnesium (Mg)	g	-	2,270	-	2,270	-	780	-
Lead (Pb)	g	-	56	-	56	-	104	-
Zinc (Zn)	g	-	3,570	-	3,570	-	2,310	-
Total Phosphorus	g	-	8,765	-	8,765	-	1,545	-
Total Nitrogen	g	-	13,250	-	13,250	-	7,650	-
Potassium	g	-	895	-	895	-	271	-
Waste								
Waste Sludge	kg	0.22	54.41	0.26	54.41	*avoided*		-
Distance to dumpsites	km	60	60	60	60	-		-
Transportation, EURO 4	kgkm	12.90	3,265	15.60	3,265	-		-

Table S4. Detailed life cycle inventory for the Philippine Electricity Mix (Department of Energy, 2019)

**Products**		
Electricity mix, PH	100	MJ
**Electricity/heat**		
Nonrenewable energy sources	**70.5**	**%**
Electricity production, Anthracite coal	14.37	MJ
Electricity production, Bituminous coal	14.37	MJ
Electricity production, Lignite coal	14.37	MJ
Electricity production, Natural gas	12.9	MJ
Electricity production, Diesel	7.25	MJ
Electricity production, Residual fuel oil	7.25	MJ
Renewable Energy Sources	**29.5**	**%**
Electricity production, Hydroelectric power plant	13.6	MJ
Electricity production, Solar	5.45	MJ
Electricity production, Wind power at wind turbine	1.65	MJ
Electricity production, geothermal	7	MJ
Electricity production, biomass	1.8	MJ
**Emissions to air**		
Heat, waste	18.9	MJ

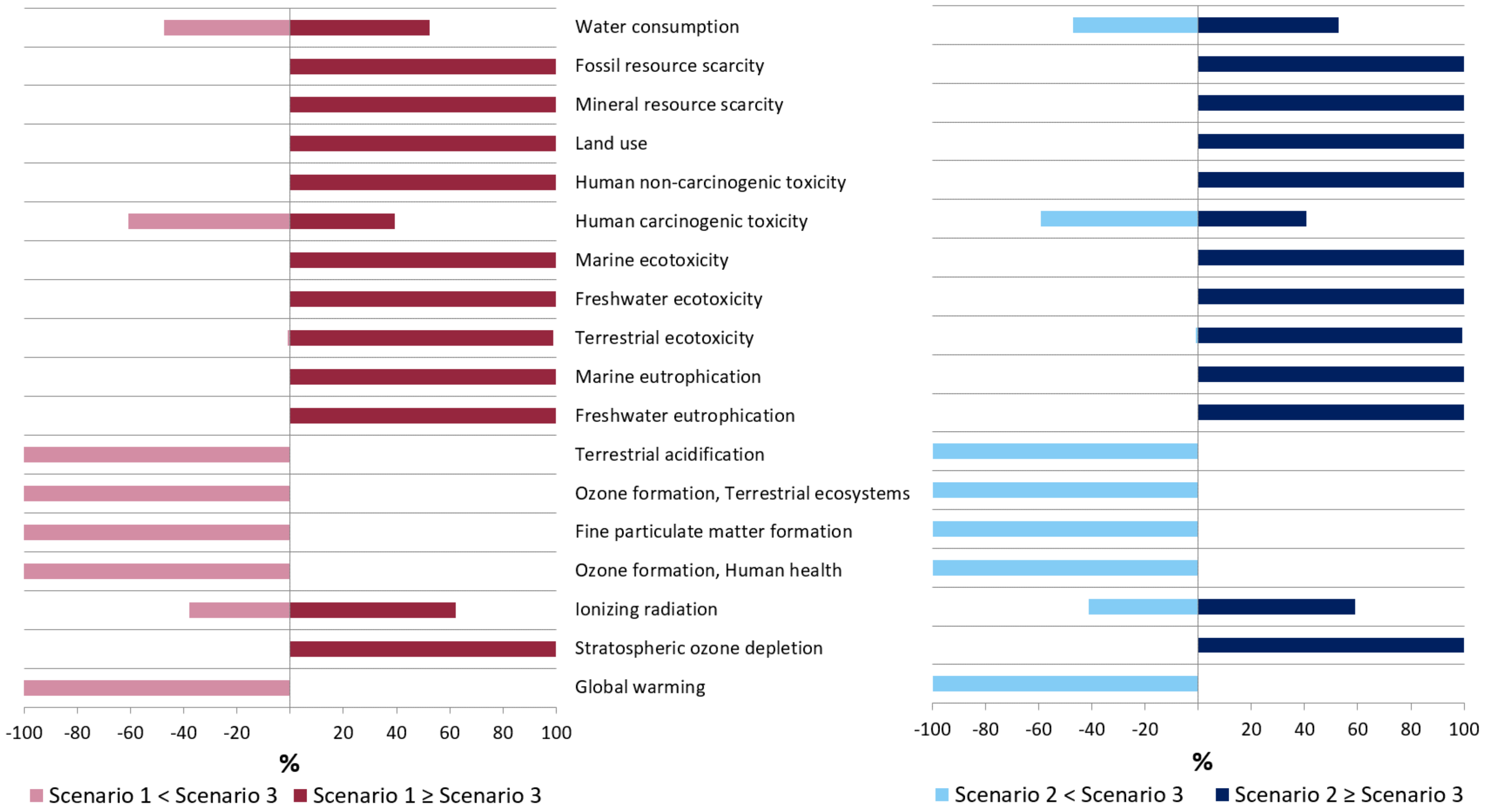

Figure S2. Detailed uncertainty analysis results for a) Scenario 1 and Scenario 3; and b) Scenario 2 and Scenario 3

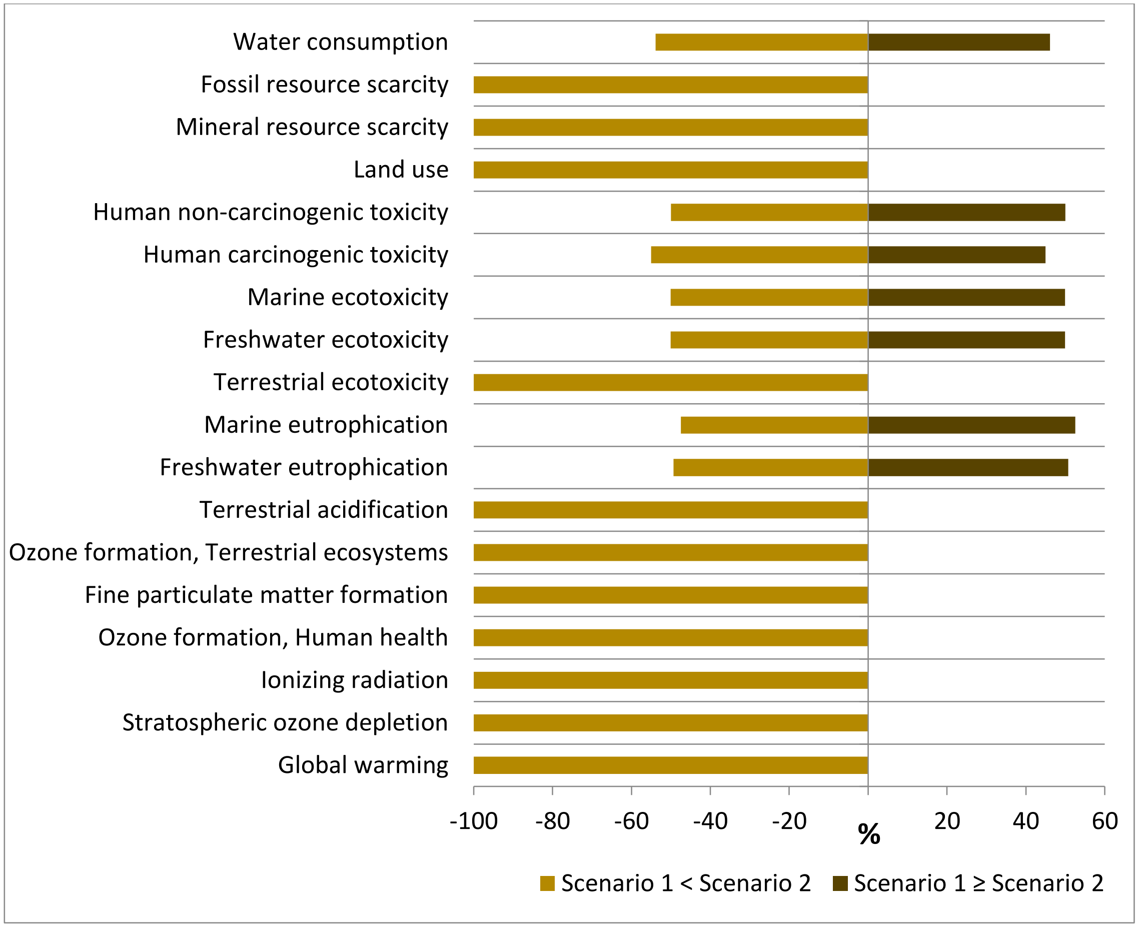

Figure S3. Detailed uncertainty analysis results for Scenario 1 and Scenario 2

Table S5. Sensitivity analysis runs for electricity mix

**Products**																						
Electricity mix, AC, consumption mix, at consumer, < 1kV/PH	100	MJ	**Sensitivity Analysis Runs**																			
**Electricity/heat**			**1**	**2**	**3**	**4**	**5**	**6**	**7**	**8**	**9**	**10**	**11**	**12**	**13**	**14**	**15**	**16**	**17**	**18**	**19**	**20**
Nonrenewable energy sources	**70.5**	**%**	**66.18**	**57.55**	**69.42**	**64.03**	**60.79**	**56.47**	**52.16**	**58.63**	**50.00**	**62.95**	**61.87**	**54.32**	**68.34**	**55.39**	**65.11**	**53.24**	**67.26**	**70.50**	**59.71**	**51.08**
Electricity production, Anthracite coal	14.37	MJ	13.65	12.21	14.19	13.29	12.75	12.03	11.31	12.39	10.95	13.11	12.93	11.67	14.01	11.85	13.47	11.49	13.83	14.37	12.57	11.13
Electricity production, Bituminous coal	14.37	MJ	13.65	12.21	14.19	13.29	12.75	12.03	11.31	12.39	10.95	13.11	12.93	11.67	14.01	11.85	13.47	11.49	13.83	14.37	12.57	11.13
Electricity production, Lignite coal	14.37	MJ	13.65	12.21	14.19	13.29	12.75	12.03	11.31	12.39	10.95	13.11	12.93	11.67	14.01	11.85	13.47	11.49	13.83	14.37	12.57	11.13
Electricity production, Natural gas	12.9	MJ	12.18	10.74	12.72	11.82	11.28	10.56	9.84	10.92	9.48	11.64	11.46	10.20	12.54	10.38	12.00	10.02	12.36	12.90	11.10	9.66
Electricity production, Diesel	7.25	MJ	6.53	5.09	7.07	6.17	5.63	4.91	4.19	5.27	3.83	5.99	5.81	4.55	6.89	4.73	6.35	4.37	6.71	7.25	5.45	4.01
Electricity production, Residual fuel oil	7.25	MJ	6.53	5.09	7.07	6.17	5.63	4.91	4.19	5.27	3.83	5.99	5.81	4.55	6.89	4.73	6.35	4.37	6.71	7.25	5.45	4.01
Renewable Energy Sources	**29.5**	**%**	**33.82**	**42.45**	**30.58**	**35.97**	**39.21**	**43.53**	**47.84**	**41.37**	**50.00**	**37.05**	**38.13**	**45.68**	**31.66**	**44.61**	**34.89**	**46.76**	**32.74**	**29.50**	**40.29**	**48.92**
Electricity production, Hydroelectric power plant	13.6	MJ	14.46	16.19	13.82	14.89	15.54	16.41	17.27	15.97	17.70	15.11	15.33	16.84	14.03	16.62	14.68	17.05	14.25	13.60	15.76	17.48
Electricity production, Solar	5.45	MJ	6.31	8.04	5.67	6.74	7.39	8.26	9.12	7.82	9.55	6.96	7.18	8.69	5.88	8.47	6.53	8.90	6.10	5.45	7.61	9.33
Electricity production, Wind power at wind turbine	1.65	MJ	2.51	4.24	1.87	2.94	3.59	4.46	5.32	4.02	5.75	3.16	3.38	4.89	2.08	4.67	2.73	5.10	2.30	1.65	3.81	5.53
Electricity production, geothermal	7	MJ	7.86	9.59	7.22	8.29	8.94	9.81	10.67	9.37	11.10	8.51	8.73	10.24	7.43	10.02	8.08	10.45	7.65	7.00	9.16	10.88
Electricity production, biomass	1.8	MJ	2.66	4.39	2.02	3.09	3.74	4.61	5.47	4.17	5.90	3.31	3.53	5.04	2.23	4.82	2.88	5.25	2.45	1.80	3.96	5.68

Table S6. Endpoint damage indicator characterisation or aggregation for all scenarios

**Endpoint level damage indicators**	**Unit**	**Scenario 1**	**Scenario 2**	**Scenario 3**
Human health	DALY	0.0237	0.0238	0.0155
Ecosystem	species-yr	5.26×10^{-07}	5.41×10^{-07}	3.16×10^{-07}
Resources	USD2013	0.1371	0.2663	-5.7670

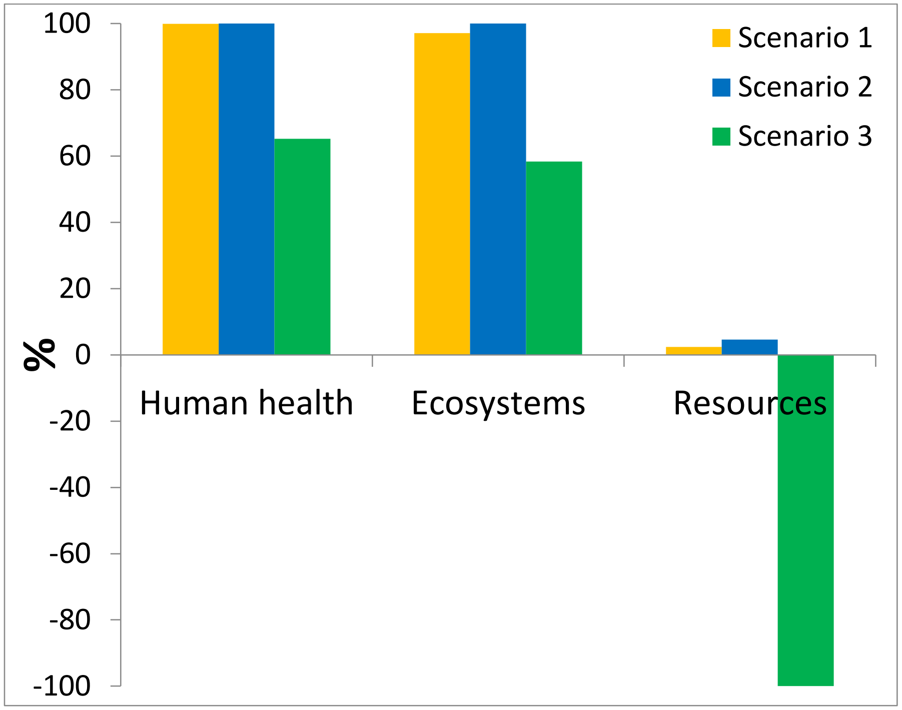

Figure S4. Environmental impact assessment-endpoint damage level normalisation for Scenario 1, Scenario 2, and Scenario 3
